# Development of an immunochromatographic strip for rapid detection of H7 subtype avian influenza viruses

**DOI:** 10.1186/s12985-021-01537-9

**Published:** 2021-04-07

**Authors:** Ge Li, Xun Wang, Qingmei Li, Jifei Yang, Xiao Liu, Wenbao Qi, Junqing Guo, Ruiguang Deng, Gaiping Zhang

**Affiliations:** 1 College of Veterinary Medicine, Henan Agricultural University, Zhengzhou, 450002 China; 2 Key Laboratory of Animal Immunology, Henan Academy of Agricultural Sciences, Zhengzhou, 450002 China; 3 College of Veterinary Medicine, South China Agricultural University, Guangzhou, 510000 China; 4 Jiangsu Co-Innovation Center for the Prevention and Control of Important Animal Infectious Disease and Zoonoses, Yangzhou University, Yangzhou, 225009 China

**Keywords:** Avian influenza virus, Rapid detection, H7 subtype, Monoclonal antibodies, Immunochromatographic strip

## Abstract

**Background:**

H7N9 avian influenza virus (AIV) including highly and low pathogenic viruses have been detected in China since 2013. H7N9 AIV has a high mortality rate after infection in humans, and most human cases have close contacted with poultry in the live poultry market. Therefore, it is necessary to develop a rapid point-of-care testing (POCT) technique for H7N9 AIV detection.

**Methods:**

The H7N9 AIV was inactivated and purified, and was used as the antigen to immunize BALB/c. Twelve H7-HA specific monoclonal antibodies (McAbs) were produced through the hybridoma technique. The McAb 10A8 was conjugated with colloid gold as detecting antibody; McAb 9B6 was dispensed on the nitrocellulose membran as the capture test line and the Goat-anti mouse IgG antibody was dispensed as control line respectively. The immunochromatographic strip was prepared.

**Results:**

The analysis of ELISA and virus neutralization test showed that the obtained McAbs specifically recognized H7 HA. Based on the prepared strip, the detection of H7 AIV was achieved within 10 min. No cross-reaction occurred between H7 AIVs and other tested viruses. The detection limit of the strip for H7 was 2.4 log_10_EID_50_/0.1 mL for chicken swab samples.

**Conclusion:**

The McAbs were specific for H7 and the immunochromatographic strip developed in this study was convenient, rapid and reliable for the detection of H7 AIV. The strip could provide an effective method for the rapid and early detection of H7 AIV.

## Introduction

Human infected with H7N9 avian influenza virus (AIV) was first reported in the spring of 2013 in China [1, 2]. As of 24th June 2019, a total of 1568 laboratory-confirmed human cases and at least 615 related deaths have been reported [3, 4]. The main source of these human cases is thought to be infected live birds or contaminated environments, particularly in live poultry markets [5, 6]. During the fifth wave of epidemics, the H7N9 AIV was genotyped into two independent lineages, the Yangtze River Delta lingage and the Pearl River Delta lineage [4]. Highly pathogenic (HP)-H7N9 variants appeared during the 5th wave, in which the isolates had 2–3 additional basic amino acid residues insertion at the hemagglutinin (HA) cleavage site (CS) [7–10], resulting in high morbidity and mortality among poultry. The highly pathogenic H7N9 virus has posed a serious threat to public health and poultry farming [4].

Early diagnosis and management are crucial to controlling H7N9 infection. Therefore, it is necessary to develop a rapid point-of-care testing (POCT) technique for H7N9 AIV detection. Serological and molecular methods have been used for detecting H7N9 AIVs [11, 12]. The National Avian Influenza Reference Laboratory (NAIRL) has established serological diagnostic techniques including hemagglutination (HA) and hemagglutination inhibition (HI) assays, agar gel immunodiffusion (AGID) assays, neuraminidase inhibition (NI) assays and indirect enzyme-linked immunosorbent assays (ELISA). Molecular diagnostic techniques include reverse transcription-polymerase chain reaction (RT-PCR) and real-time RT-PCR [13, 14]. However, these traditional detection methods are not only time-consuming, laborious with complicated operations, but also prone to false positive results. In addition, traditional diagnostic methods usually require special equipment, which limits the rapid detection for large number of samples. Compared with other detection methods, the immunochromatographic test strip labled with colloidal gold is more attractive because it is rapid and does not require extra equipment for detection [15].

Therefore, in this study McAbs were prepared using the inactivated H7N9 virus as an immunogen. An immunochromatographic strip specific for AIV H7 subtype was then developed using two H7-HA specific McAbs, which can detect clinical samples within 10 min with high specificity and sensitivity.

## Materials and methods

### Viruses

The H7N9 AIVs including the HP-H7N9 AIV (A/Chicken/Huizhou/HZ-3/2016), the LP-H7N9 AIV (A/Chicken/Guangdong /G1/2013), the LP-H7N9 AIV (A/Chicken/Guangdong /SW154/2015), A/Guangdong/GH0741/2013, and other subtype AIVs including A/Swine/Guangxi/NN1994/2013 (H1N1), A/Swine/Guangxi/NNXD/2016 (H3N2), A/Duck/Yunnan/YN-9/2016 (H5N6) and A/Chicken/Guangdong/V/2008 (H9N2) were provided by the BSL3 Laboratory at South China Agricultural University.

The H7N9 AIVs including A/Chicken/Jiangsu/JX148/2014, A/Chicken/Jiangsu/JT98/2014, A/Chicken/Jiangsu/WJ170/2014, A/Chicken/Jiangsu/TM103/2014, A/Chicken/Shandong/SDL101/2014, A/Chicken/Jiangsu/JT115/2015, A/Chicken/Jiangsu/XZ256/2015, A/Chicken/Zhejiang/JX158/2015, A/Chicken/Anhui/AH284/2015, A/Chicken/Jiangsu/RG126/2015, A/Chicken/Shandong/SD183/2016, A/Chicken/Jiangsu/JS11/2016, A/Chicken/Jiangsu/JT156/2016, A/Chicken/Liaoning/LN1/2016, A/Chicken/Guangdong/GD15/2016, A/Chicken/Zhejiang/ZJ19/2017, A/Chicken/Jiangsu/LY246/2017, A/Chicken/Jiangsu/0116/2017, A/Chicken/Jiangsu/JT186/2017 and A/Chicken/Guangdong/GD4/2017 were provided by the College of Veterinary Medicine, Yangzhou University.

The other avian viruses such as avian infectious bronchitis virus (IBV), Newcastle disease virus (NDV), Marek’s disease virus (MDV), and avian infectious bursal disease virus (IBDV) were obtained from the Key Laboratory of Animal Immunology, Henan Academy of Agricultural Sciences, China.

Antigen strains of H7-Re2 and H7-Re3 were provided by State Key Laboratory of Veterinary Biotechnology, Harbin Veterinary Research Institute, Chinese Academy of Agricultural Sciences, Harbin, China.

### Monoclonal antibodies production

McAbs against H7N9 were developed following a standard procedure. Six-weeks-old female BALB/c mice were immunized with the inactivated H7N9 AIV (A/Chicken/Huizhou/HZ-3/2016) purified by differential centrifugation at an immunization dose of 20 μg/mouse in Freund’s adjuvant twice with a 3-week interval followed by final immunization with 20 μg H7N9 antigen at 3 days before cell fusion. Splenocytes from the immunized mouse were fused with Sp2/0 myeloma cells, and the hybridoma cells were screened by immunoperoxidase monolayer assay (IPMA) and enzyme-linked immunosorbent assay (ELISA) and cloned by the limiting dilution method. The ascitic fluids from the positive hybridomas were produced in mice.

### Screening antibodies specific for HA protein

McAbs against the HA protein of H7 subtype AIV were screened by ELISA. The HA proteins of different influenza virus subtypes (Table 1) diluted in carbonate buffer (CBS) at a concentration of 1 μg/mL were added into 96-well plates at 50 μL/well and incubated at 37 °C for 2 h. After blocked with 5% skim milk at 37 °C for 1 h; hybridoma supernatant of McAbs were added and incubated at 37 °C for 30 min. The reactions were then detected by HRP-labled Goat anti-mouse IgG, and color was developed using TMB solution at room temperature for 10 min which was was stopped by stop solution. The OD_450_ value of each well was read with a microplate reader for statistical analysis.

**Table 1:** Recombinant HA proteins of influenza virus

Subtype	Recombinant HA protein^a^
H1N1	A/California/04/2009
H3N2	A/California/7/2004
H5N1	A/Auhui/1/2005
H7N9	A/Anhui/1/2013
H9N2	A/Hong Kong/1073/99

^a^Recombinant HA protein were bought from Sino Biological Inc (Beijing, China)

### Identification of antigen epitopes recognized by monoclonal antibodies

The peptide scanning technique was used to identify the epitope recognized by the McAbs. According to the H7N9 subtype avian influenza HA protein amino acid sequence (ARG44098.1), peptide was synthesized by GL Biochem Ltd (Shang hai, China). The peptide was coupled to the bovine serum albumin (BSA) carrier protein by Sulfo-SMCC, and the coupled peptide was spot-printed on the nitrocellulose membrane. The H7N9 positive serum was used as a positive control, and BSA was used as a negative control. The supernatant of McAbs 9B6 and 10A8 were used as primary antibody, and reactions were then detected by HRP-labled Goat anti-mouse IgG. Finally, ECL color reagent was used to detect the reactivity of McAbs and HA polypeptide.

### Virus neutralization test

Neutralizing activities of McAbs were determined by HI assay and virus neutralization (VN) assays. Briefly, tenfold serial dilutions of McAbs from 10^3^ were mixed with 200 TCID_50_ virus and incubated for 2 h at 37 °C. The mixture was then used to infect Madin-Daby canine kidney cells (MDCK) and incubated for 24 h at 37 °C. Then the 100% VN titers of McAbs were determined by Reed-Muench. At the same time, tenfold serial dilutions of McAbs were mixed with virus to determine HI titers, and HI titers ≥ 4 were considered positive.

### Preparation of colloidal gold and gold-labeled antibodies

Preparation of colloidal gold by trisodium citrate method [16]. Briefly, 1 mL of 1% chloroauric acid and 99 mL of double distilled water was added into the erlenmeyer flask, stiring and heating, followed by the rapid addition of 1.6 mL of 1% trisodium citrate solution with rapid stirring. The reaction mixture was boiled until the color gradually changes from light yellow to deep red and no longer changes in color, with the above process taking about 20 min. The colloidal gold solution was cooled to room temperature. 12 McAbs were incubated with different pH colloidal gold solution for 30 min. The 10% BSA was added to the colloidal gold conjugation and incubated for 10 min. The mixture was then centrifuged at 13,000 rpm and 4 °C for 30 min to remove any unbound antibody. The pellet was resuspended in boric acid buffer containing 1% BSA.

### Selection of paired McAbs for the strip

Among the twelve positive clones, two H7-HA McAbs which showing higher binding affinity were selected to establish a rapid detective strip by sandwich Dot-blot. The sandwich Dot-blot was performed as following. Twelve capture antibodies was blotted on the nitrocellulose membrane (Table 2) at 37 °C for 30 min. After blocking the nitrocellulose membrane using phosphate buffered solution (PBS) containing 1% BSA, 200 μL per membrane of sample diluted in antigen dilution buffer were added and then incubated for 30 min. Then the membrane were rinsed five times with PBS containing 0.2% Tween 20. Twelve colloidal gold conjugated McAbs was added to twelve membranes with 50 μL every membrane, respectively. The pairing of two specific antibodies were selected by observing the color strength of the nitrocellulose membrane.

**Table 2:** Dot-Blot layout of 12 monoclonal antibodies

Antibody number	a	b	c	d
1	2A2	3E5	3H3	7H7
2	11B8	10A8	9B6	12F11
3	13C10	15C5	16D2	21E2

### Preparation of the rapid detective strip

Two McAbs with good specificity were selected to develop the immunochromatographic strip. Briefly, the purified H7-HA specific McAbs were labeled with colloidal gold as conjugated mAb then dispensed on the fiberglass pads to generate conjugate pads. The conjugate pad was dried at 42 °C for 50 min. On a 2.79-cm nitrocellulose membrane, the H7-HA specific McAbs and rabbit anti-mouse IgG antibody solutions were dispensed as test and control lines, respectively. The nitrocellulose membrane was dried at 45 °C for 4 h. The fiberglass sample pad, conjugate pad, nitrocellulose membrane, and absorption pad were assembled on the support board sequentially, with 1–2 mm overlapping each other and cut into 2.79-mm pieces (CM 4000 cutter; Bio-Dot).

### Broad reaction of the strip for H7 subtype AIVs

To evaluate the broad reaction of the rapid detective strip, H7N9 AIVs isolated from 2013 to 2017 and antigen strains including H7-Re-2 and H7-Re-3 were tested. 100 μL of each sample cotaining 10^5^ TCID_50_ allantoic fluid or original solution of H7-Re-2 and H7-Re-3 were added to the sample pad of the test strip and incubated for 10 min at room temperature.

#### Specificity evalution of the rapid detective strip

To evaluate the specificity of the rapid detective strip, the H1, H3, H5, H7, H9 subtype influenza viruses and other avian viruses including NDV, MDV, IBV and IBDV were simultaneously detected. 100 μL of each sample containing 10^5^ TCID_50_ virus was added to the sample pad of the test strip and incubated for 10 min at room temperature.

#### Sensitivity evalution of the Rapid rapid detective strip

Three viruses HZ-3, G1 and SW15154 of the H7 subtype were used to detect the sensitivity of the rapid detection strip. The virus was diluted 2 times with 0.01 M PBS from 2^−1^ to 2^−15^ and PBS was used as a negative control.

#### Stability evalution of the rapid detective strip

These strips were tested to determine their sensitivity in detecting the virus HZ-3 upon storage at room temperature for 6 months. The virus HZ-3 was diluted 2 times with 0.01 M PBS from 2^−1^ to 2^−15^ and PBS was used as a negative control.

#### Detecting tissue samples from experimentally infected chickens

Three-weeks-old SPF chickens purchased from Beijing Boehringer Ingelheim Vital Biotechnology Co., Ltd, were inoculated intranasally with 10^6^ EID_50_ of H7N9 AIV (A/Chicken/Huizhou/HZ-3/2016) in a 0.2 mL volume (n = 6). In addition, the other two chickens were not inoculated with virus as negative control. After 60 h, the virus-infected chickens began to die. In order to confirm H7N9 AIV infection, the tissues (brain, windpipe, heart, liver, spleen, lung, thymus, pancreas, bursa of fabricius and cecal tonsil) were dissected from each chicken, and these samples were tested using the H7 detection strip and RT-PCR, respectively.

#### Detection of simulated clinical swab samples

Tracheal swabs and cloacal swabs (n = 30) were collected from healthy poultry in Henan Province. Swab samples were collected in 2 mL PBS, and the virus allantoic fluids (HZ-3) were added into tracheal swabs and cloacal swabs to simulate clinical swab samples. The simulated clinical swab samples were 2-time-diluted from 2^−8^ to 2^−12^ to evaluate the rapid detection strip by HA test and strip test.

## Results

### Preparation and characterization of monoclonal antibodies

**Fig. 1:**
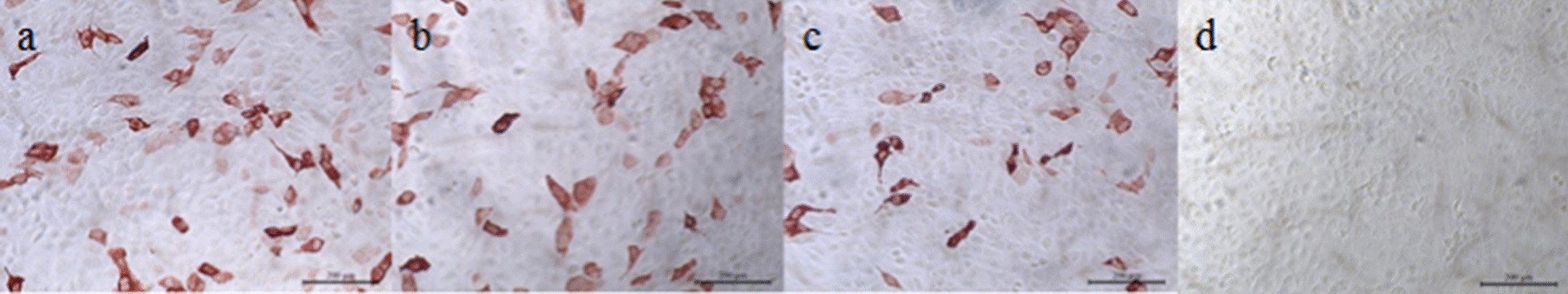
IPMA for H7-HA specific McAbs. **a** 9B6; **b** 10A8; **c** mouse positive serum; **d** mouse negative serum

Twelve McAbs against H7N9 AIVs HA protein were produced, and only two McAbs 9B6 and 10A8 related to the strip were introduced in this article (Fig. 1). The IPMA titers of both two McAbs were 10^−6^. The HI titers of the two McAbs were 13 log2 and 4 log2, and 100% VN titers of the two McAbs were 1:16 000 and 1:1 000, respectively. The epitopes recognized by McAbs 9B6 and 10A8 were determined as GVTSACRRSGSSFYAEMK (aa positions 142–159), and YKSTQSAIDQITGKLNRL (aa positions 381–398), respectively (Table 3).

**Table 3:** Characterization of H7-HA specific McAbs generated in this study

Name	Isotype	IPMA titer	HI titer (log2)	100% VN titer	Epitope
9B6	IgG1	10^−6^	13	1:16 000	GVTSACRRSGSSFYAEMK
10A8	IgG2a	10^−6^	4	1:1 000	YKSTQSAIDQITGKLNRL

### Establishment of a rapid detective immunochromatographic strip

**Fig. 2:**
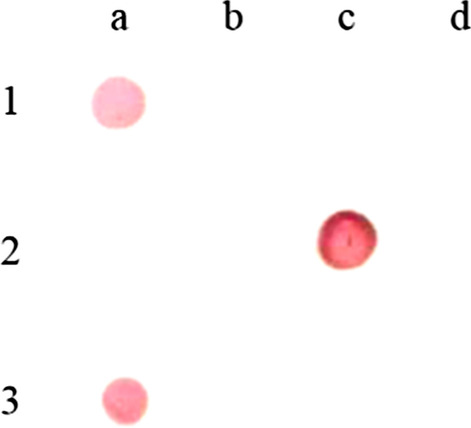
Dot-Blot of paired McAbs. Conjugated antibody 10A8 paired with twelve different McAbs which were dispensed on the nitrocellulose membrane (1A-3D). 1a–d: McAbs 2A2, 3E5, 3H3 and 7H7; 2a–d: 11B8, 10A8, 9B6 and 12F11; 3a–d: 13C10, 15C5, 16D2 and 21E2

The colloidal gold was obtained and labeled with twelve McAbs. After several comparable experiments, the optimum pH was found to be 4, and the optimum labeled McAbs dose was 4 μg/mL. It was validated that the McAb 9B6 used as capture antibody and McAb 10A8 used for detection showed the best performance (2c) in this assay (Fig. 2). The colloidal gold conjugation of 10A8 was dispensed on the fiberglass pads as conjugated McAb. The dosage of colloidal gold-labeled 10A8 antibody mixture was 1.79 μL/cm. The McAb 9B6 was diluted to 0.8 mg/mL in physiological saline and dispensed on the nitrocellulose membran as the capture test line. Then the Goat-anti mouse IgG antibody was diluted to 1.3 mg/mL in physiological saline as control line to develop the immunochromatographic strip.

### Broad reaction of the strip for H7 subtype AIVs

**Fig. 3:**
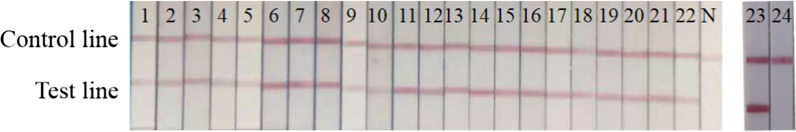
Broad reaction of the rapid detective strip for H7 subtype AIVs. 1: A/Chicken/Guangdong/G1/2013; 2: A/Guangdong/GH0741/2013; 3: A/Chicken/Jiangsu/JX148/2014; 4: A/Chicken/Jiangsu/JT98/2014; 5: A/Chicken/Jiangsu/WJ170/2014; 6: A/Chicken/Jiangsu/TM103/2014; 7: A/Chicken/Shandong/SDL101/2014; 8: A/Chicken/Jiangsu/JT115/2015; 9: A/Chicken/Jiangsu/XZ256/2015; 10: A/Chicken/Zhejiang/JX158/2015; 11: A/Chicken/Anhui/AH284/2015; 12: A/Chicken/Jiangsu/RG126/2015; 13: A/Chicken/Shandong/SD183/2016; 14: A/Chicken/Jiangsu/JS11/2016; 15: A/Chicken/Jiangsu/JT156/2016; 16: A/Chicken/Liaoning/LN1/2016; 17: A/Chicken/Guangdong/GD15/2016; 18: A/Chicken/Zhejiang/ZJ19/2017; 19: A/Chicken/Jiangsu/LY246/2017; 20: A/Chicken/Jiangsu/0116/2017; 21: A/Chicken/Jiangsu/JT186/2017; 22: A/Chicken/Guangdong/GD4/2017; N: PBS; 23: H7-Re2; 24: H7-Re3

H7N9 viruses isolated from 2013 to 2017 and antigen strain H7-Re2 showed two red lines in the test control area, indicating that the strip could detect H7 subtype AIVs with broad reaction (Fig. 3). Antigen strain H7-Re3 showed one red line in the test control area.

### Specificity evaluation of the rapid detective strip

**Fig. 4:**
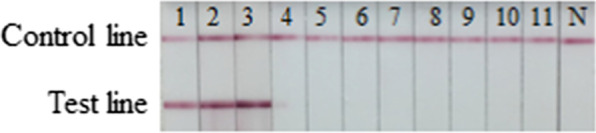
Specificity of the rapid detective strip. 1: A/Chicken/Huizhou/HZ-3/2016 (H7N9); 2: A/Chicken/Guangdong/G1/2013 (H7N9); 3: A/Chicken/Guangdong/SW154/2015 (H7N9); 4 A/Swine/Guangxi/NN1994/2013 (H1N1); 5: A/Swine/Guangxi/NNXD/2016 (H3N2); 6: A/Duck/Yunnan/YN-9/2016 (H5N6); 7: A/Chicken/Guangdong/V/2008 (H9N2); 8: NDV; 9: MDV; 10: IBV; 11: IBDV; N: PBS

A rapid detective strip for the double antibody sandwich mode was established using H7-HA specific mAbs 9B6 and 10A8 as capture and conjugation antibodies, respectively. The specificity test results showed that only H7 subtype had two red bands at the T and C lines, and other subtypes of influenza virus and poultry virus had only one red band at the C line (Fig. 4), indicating that the rapid detective strip had high specificity for the detection of H7 AIVs.

### Sensitivity evaluation of the rapid detective strip

**Fig. 5:**
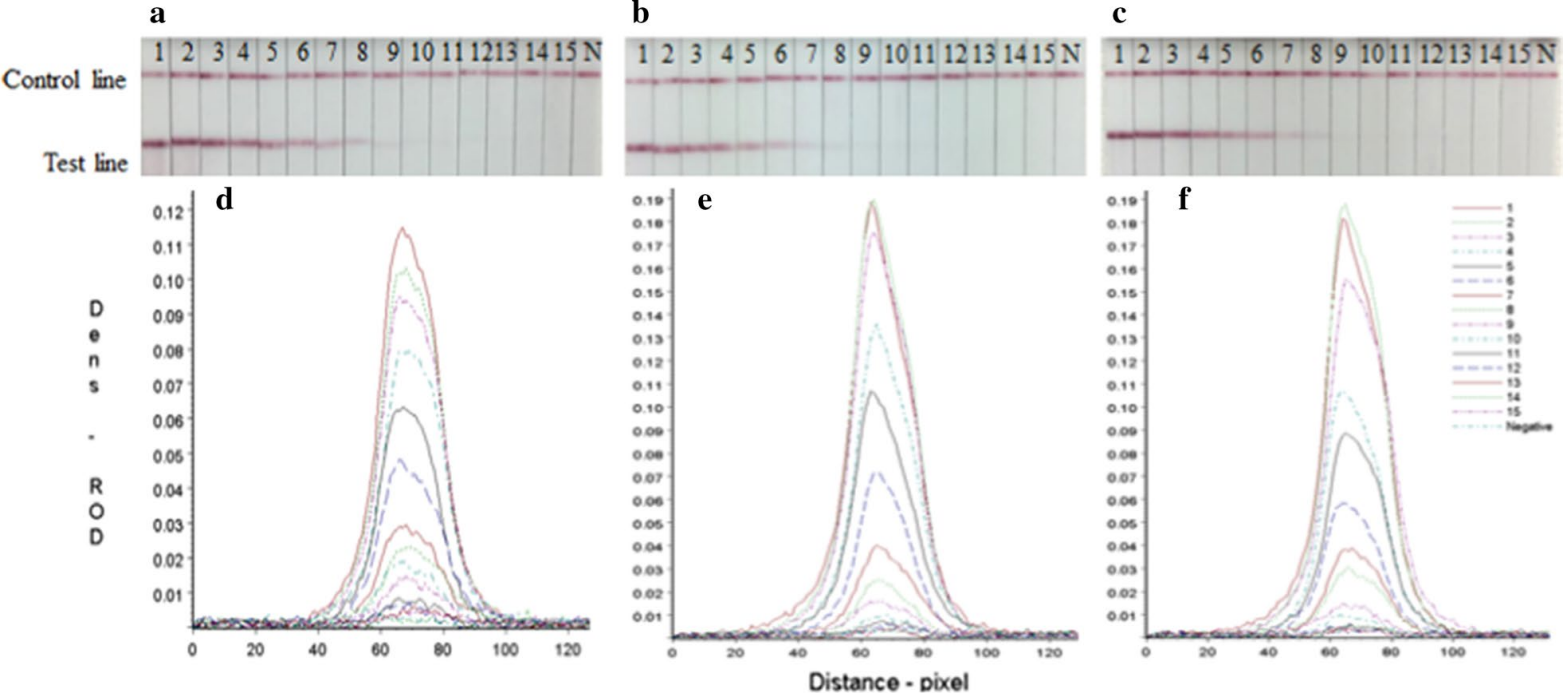
Sensitivity of the rapid detective strip. **a, b** and **c**. 1–15: Diluted positive sample ranging from 2^−1^ to 2^−15^ **a** HP-H7N9 AIV (A/Chicken/Huizhou/HZ-3/2016), **b** the LP-H7N9 AIV (A/Chicken/Guangdong /G1/2013), **c** the LP-H7N9 AIV (A/Chicken/Guangdong /SW154/2015). N:PBS negative control. **d**, **e** and **f** The colored membranes were screened under a TSR-3000 Reader, and relative optical density (ROD) values were analyzed by AIS software

The sensitivity of the rapid detective strip was tested using three strains of H7 AIVs (HZ-3, G1 and SW15154). The results of the strip were read by TSR-3000 Reader showed that the sensitivity of the strip could be 2^−11^ (Fig. 5), which was 2–8 times higher than the HA titer of chicken embryo allantoic fluid (Table 4), and 10^2.6^ TCID_50_ or 10^2.4^ EID_50_ could be detected by using the strip, indicating that the rapid detection strip had high sensitivity for the detection of H7 AIVs.

**Table 4:** Sensitivity evaluations of the rapid detection method

Virus	Name	Virus titer		HA titer	Strip test	
		(EID_50_/0.1 mL)	(TCID_50_/0.1 mL)		Results	G/D × area-ROD (pixel)
A-HP-H7N9	HZ-3^a^	10^5.7^	10^5.9^	2^−8^	2^−11^	11.50
B-LP-H7N9	G1^b^	10^5.5^	10^6.1^	2^−9^	2^−9^	14.03
C-LP-H7N9	SW15154^c^	10^5.7^	10^5.9^	2^−8^	2^−9^	14.57

^a^HP-H7N9 AIV (A/Chicken/Huizhou/HZ-3/2016)

^b^The LP-H7N9 AIV (A/Chicken/Guangdong /G1/2013)

^c^The LP-H7N9 AIV (A/Chicken/Guangdong /SW154/2015); HA = hemagglutination; PBS = phosphate-buffered saline: G/D × area − ROD (pixel) = graph density × area-relative optical density

### Stability evaluation of the rapid detective strip

**Fig. 6:**
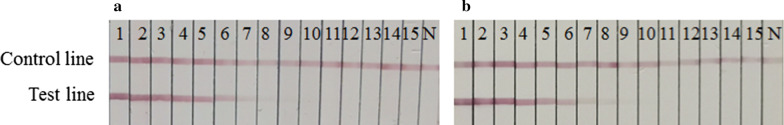
Stability of the rapid detective strip. **a** The sensitivity of fresh strips were determined; **b** Strips after 6 months of storage were determined. **a** and **b** 1–15: Diluted positive sample ranging from 2^−1^ to 2^−15^ the LP-H7N9 AIV (A/Chicken/Guangdong /G1/2013). N: PBS negative control

The strips still had the same detection limit for H7 AIV (A/Chicken/Guangdong/G1/2013) as freshly produced strips after 6 months of storage, indicating that the gold immunochromatographic strip had high sensitivity for the detection of H7 AIVs (Fig. 6).

### Detection of tissue samples from infected chickens

RT-PCR test and strip test were performed on 60 infected tissue samples and 20 uninfected tissue samples, and the strip test results were compared with the RT-PCR test results. The results of the RT-PCR test showed that 100% (60/60) of infected tissue samples were positive for H7N9 AIV and 100% (20/20) of uninfected tissue samples were negative for H7N9 AIV, consistent with 100% (60/60) of infected tissue samples were positive for H7N9 AIV and 100% (20/20) of uninfected tissue samples were negative for H7N9 AIV of determined by strip test (Table 5).

**Table 5:** Detection of H7N9 AIV antigen in the infected or uninfected chickens

Sample	RT-PCR		Strip		Coincidence %
	Positive	Negative	Positive	Negative	
Infected chicken 1	10	0	10	0	100
Infected chicken 2	10	0	10	0	100
Infected chicken 3	10	0	10	0	100
Infected chicken 4	10	0	10	0	100
Infected chicken 5	10	0	10	0	100
Infected chicken 6	10	0	10	0	100
Uninfected chicken 1	0	10	0	10	100
Uninfected chicken 2	0	10	0	10	100

### Detection of simulated clinical swab samples

**Fig. 7:**
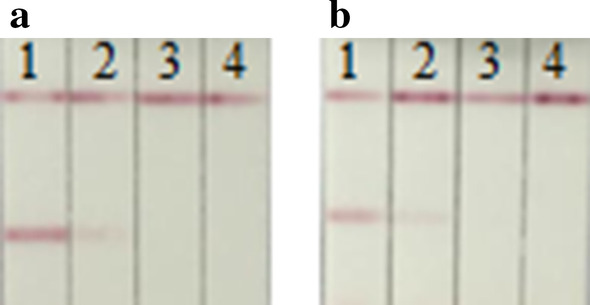
Use of the strip for detecting tracheal swabs (**a**) and cloacal swabs (**b**) from simulated clinical swab samples. a-1 and b-1: H7 positive AIV, a-2: tracheal swab sample contained 2.4 EID_50_, a-3: tracheal swab sample contained 1.4 EID_50_, a-4: PBS, b-2: cloacal swab sample contained 2.4 EID_50_, b-3: cloacal swab sample contained 1.4 EID_50_, and b-4: PBS

HA test and strip test were performed on 30 simulated clinical swab samples, and the HA test results were compared with the strip test results. The results of the HA test showed that 90% (27/30) of swab samples were positive for H7N9 AIV, consistent with 96.7% (29/30) of determined by strip test. And the results of the HA test showed that virus titers ranged from 2^−6^ to 2^−8^ in the swabs. The detection limits in both tracheal swabs and cloacal swabs in the strip test were 2^−11^ (Fig. 7). These data suggested that the rapid detection method was suitable for detecting H7N9 AIVs from infected samples.

## Discussion

With the vigorous development of the breeding industry, bird flu has always been a threat to our country's poultry breeding industry, and the requirements for the prevention and control of bird flu have become more stringent. Influenza virus is a segmented single negative-stranded RNA virus with an envelope, which is prone to mutation [7]. Nowadays, the classic and commonly used methods for detecting influenza virus include hemagglutination inhibition test and fluorescent quantitative PCR [17]. However, the use of the above methods or the need for detection equipment, or the need to prepare fresh red blood cells, and has certain requirements for operation, is not convenient for large-scale rapid screening and testing of grassroots enterprises and institutions or farmers. Therefore, colloidal gold test strips have been widely used due to their huge advantages.

The biological characteristics of monoclonal antibodies are a key factor in determining the performance of colloidal gold immunochromatography strip. In this study, we developed a rapid detective immunochromatographic strip specific for the H7 subtype by using two H7-HA specific McAbs 9B6 and 10A8 as capture and conjugation antibodies. In order to improve the specificity and affinity of McAbs, the McAbs that targeted HA protein were selected develop a sandwish mode. Two H7-HA McAbs were developed, and identified by HI assay and VN assay. The HI and VN titers of different McAbs [18] were different, which may lead to differences in the specificity and affinity of McAbs. In addition, we identified the epitopes recognized by these two McAbs through peptide scanning technology. Among them, the McAb 9B6 recognized conservative epitope GVTSACRRSGSSFYAEMK that has been reported [19], and the McAb 10A8 recognized the epitope that has been reported YKSTQSAIDQITGKLNRL [20]. The influenza strains change rapidly, and the strip composed of these two McAbs can identify H7-Re2 but not H7-Re3. The HI test and Dot test identified the reactivity of the two Mcabs with H7-Re2 and H7-Re3 respectively. The results showed that 9B6 can react with both H7-Re2 and H7-Re3, while 10A8 can only react withH7-Re2. Therefore, the strip developed in this study can be used to differentially diagnose H7-Re2 and H7-Re3 strains. In subsequent experiments, we will update the antibodies used on the test strips in time according to the variation of influenza strains to achieve the purpose of rapid detection. At the same time, different batches of ascites may affect the performance of the test strips. For this reason, we prepared a large amount of ascites at one time and purified and packed them to ensure the availability of experimental data.

The colloidal gold immunochromatography technology is convenient to apply, and the results show rapid and intuitive, rapid diagnosis of diseases, high sensitivity, and rapid application in the field of animal medicine. Manzoor [21] developed a Pensite test kit for rapid diagnosis of H7 highly pathogenic avian influenza. The detection limit for swab samples and tissue homogenates was 4.5 log10EID_50_, and the sensitivity was lower than our strips (2.4 log10EID_50_). Liu X [18] developed a colloidal gold-based immunochromatographic strip for rapid detection of H7N9 influenza viruses with a limit of detection of 2.5 log10EID_50_, less sensitive than our strips (2.4 log10EID_50_). Kang [22] developed a rapid immunochromatographic test for hemagglutinin antigen of H7 subtype in patients infected with novel avian influenza A (H7N9) virus with a limit of detection of 10^3^ TCID_50_, less sensitive than our strips (10^2.6^ TCID_50_). Although the rapid immunoassay was less sensitive than rRT-PCR or virus isolation, the strips could achieve POCT, which could be used as an indicator for H7N9 AIVs infections. The rapid spread of highly pathogenic H7N9 subtype avian influenza viruses around the world may increase the risk of human infections. H7N9 subtype AIVs isolated from human and chicken were successfully detected by the strip, indicating a broad reaction for H7N9 AIVs. Morever, the strips did not detect the other subtypes of influenza virus and poultry virus. Therefore, a rapid detection method based on double antibody sandwich mode H7 AIVs was successfully prepared in this study, which has good specificity, sensitivity and stability, and provided technical support for the diagnosis and monitoring of H7 AIVs.

## Conclusions

H7N9 infected human and chicken and most human cases have close contacted with poultry in the live poultry market. In this study, 12 H7-HA specificMcAbs were produced and two McAbs 9B6 and 10A8 were used to develop an immunochromatographic strip for detecting H7 AIVs. The obtained McAbs and strip had a great potential in application of the rapid detection and surveillance of H7 AIV.

## Data Availability

All data and materials are available to any scientist wishing to use them.

## Abbreviations

ELISA: Enzyme-linked immunosorbent assay
McAbs: Monoclonal antibodies
AIV: Avian influenza virus
POCT: Point-of-care testing
RT-PCR: Reverse transcription-polymerase chain reaction
IPMA: Immunoperoxidase monolayer assay
